# NCA-Net for Tracking Multiple Objects across Multiple Cameras

**DOI:** 10.3390/s18103400

**Published:** 2018-10-11

**Authors:** Yihua Tan, Yuan Tai, Shengzhou Xiong

**Affiliations:** National Key Laboratory of Science & Technology on Multi-Spectral Information Processing, School of Automation, Huazhong University of Science and Technology, Luoyu Road 1037, Hongshan District, Wuhan 430074, China; m201772500@hust.edu.cn (Y.T.); xiongsz@hust.edu.cn (S.X.)

**Keywords:** multi-object tracking, multi-camera, metric learning, deep learning

## Abstract

Tracking multiple pedestrians across multi-camera scenarios is an important part of intelligent video surveillance and has great potential application for public security, which has been an attractive topic in the literature in recent years. In most previous methods, artificial features such as color histograms, HOG descriptors and Haar-like feature were adopted to associate objects among different cameras. But there are still many challenges caused by low resolution, variation of illumination, complex background and posture change. In this paper, a feature extraction network named NCA-Net is designed to improve the performance of multiple objects tracking across multiple cameras by avoiding the problem of insufficient robustness caused by hand-crafted features. The network combines features learning and metric learning via a Convolutional Neural Network (CNN) model and the loss function similar to neighborhood components analysis (NCA). The loss function is adapted from the probability loss of NCA aiming at object tracking. The experiments conducted on the NLPR_MCT dataset show that we obtain satisfactory results even with a simple matching operation. In addition, we embed the proposed NCA-Net with two existing tracking systems. The experimental results on the corresponding datasets demonstrate that the extracted features using NCA-net can effectively make improvement on the tracking performance.

## 1. Introduction

With the popularization of high-definition imaging technology and the progress of artificial intelligence, intelligent video surveillance technology has attracted much attention. As one of the crucial parts of intelligent video surveillance, multi-object tracking (MOT) is of great significance in pedestrian behavior analysis, robot navigation, autonomous driving and so on. With the development of smart cities, sensor networks, especially camera networks, play key roles in public information sharing [1], so tracking across multi-camera scenarios is more critical and brings some new challenges which were also reported in the model analysis of cameras [2] and image retrieval [3]. Although a lot of methods have been proposed, multi-pedestrian tracking across multi-camera scenarios is still a difficult challenge when the video data is taken from a camera sensor network.

To promote the rapid development of multi-pedestrian tracking technology, accurately labelleing pedestrian objects in the surveillance video is crucial, which is significant for algorithm development and performance evaluation. Many researchers have made efforts to this end facilitating this research. Gray et al. assessed the performance of the appearance models for view invariant pedestrian recognition by collecting a challenging dataset which consists of 632 pedestrian image pairs from different views [4]. Nambiar et al. presented a labeled image sequence dataset (HDA) to benchmark different video surveillance algorithms [5], where around 80 persons appeared in the dataset and most of them are visible in more than one camera. Fleuret et al. collected some multi-camera sequences for developing and testing their person detection and tracking framework [6,7,8,9]. Milan et al. constructed a carefully annotated dataset for Multi-Object Tracking Challenge 2016 [10]. In addition, the datasets which provided non-overlapping multi-camera videos covering the same objects were adopted in this paper provided by Chen et al. [11] and Ristani et al. [12]. No matter which dataset is adopted, there are still some common challenges: frequent occlusion among crowds, accurate determination of the beginning and end of a trajectory, similar appearance among pedestrians, and missing details due to low resolution.

For the sake of abundant datasets, lots of excellent algorithms have been presented in the literature recently to meet the challenge of MOT. According to whether the algorithm relies on the object detection results, they can be divided into two categories: detection-based and detection-free tracking. For the first category, the researches focus on the association of objects or short object sequences. Bose et al. utilized the background subtraction on a stationary camera to form foreground blobs, which were handled as the objects in the tracking problem with an inference graph [13]. Song et al. proposed the solution based on a sequence of tracklets and dealt with the tracklet association via a stochastic graph evolution [14]. Bae et al. also sought to resolve the issue based on tracklet association by tracklet confidence on the basis of the detectability and continuity of a tracklet [15]. Xiang et al. reformed the MOT problem with the decision making strategy in Markov Decision Processes [16]. As for detection-free algorithms, only the manually marked targets in the first frame are needed. Hu et al. exploited occlusion reasoning to realize single and multi-object tracking within the framework of Bayesian state inference based on particle filtering [17]. Zhang et al. devised a new multi-object tracker without model which learned spatial constraints along with the object detectors using an online structured SVM [18]. Sclaroff et al. formulated the appearance by assembling templates which were updated online according to the detections produced by the pedestrian detector [19].

On the other hand, regardless of the tracking framework, the feature representation of the object is also very essential for multi-pedestrian tracking. For existing algorithms, motion and appearance features were most commonly used. Yoon et al. constructed a Relative Motion Network consisting of multiple relative motion models which described spatial relations among the objects [20]. Khalili et al. tried to simulate the phenomenon that the positions of the electrons around the nucleus are uncertain through passing the particles in the areas where the pedestrian comes out more likely and applied it in particle filter framework [21]. Sugimura et al. tracked a pedestrian combining the gait features and the temporal consistency of local appearance [22]. Also, color histogram was adopted as the appearance model in [23,24,25,26]. In addition, Ma et al. exploited the non-overlap HOG feature and an oriented LBP feature [27]. Tsuduki et al. developed a method of point feature tracking using SIFT [28].

For most existing tracking algorithms, no matter how subtle they are, the artificial features being incapable of effectively distinguishing different pedestrians results in poor performance of multi-pedestrian tracking. Inspired by the successful exploitation of deep learning in many applications, we seek to apply it to the multiple pedestrians tracking task. Since AlexNet obtained excellent success on the ImageNet 2012 classification challenge [29], deep convolution neural networks have been extensively applied in computer vision tasks and achieved remarkable success. Besides, AlexNet [29], VGG Net [30], Googlenet [31] and ResNet [32] have attracted wide attention. With the use of CNN, we can acquire the high-level semantic feature representations, such as 3D-features extraction within a 3D-CNN framework for video quality assessment [33]. However, different from the general tasks such as classification, segmentation and detection, our objects are all pedestrians and the number is not sure. Therefore, it is a natural choice to adopt metric learning to drive the network training.

In this paper, a method called NCA-Net is proposed to extract deep features of pedestrians across multi-camera scenarios. We successfully combine feature learning and metric learning together through the CNN. For the feature learning, we choose the structure of three alternative convolution layers and pooling layers. As to metric learning, we adopt an unbiased convolutional layer to express the Mahalanobis distance matrix, and a NCA-based cost function is derived for training. In addition, in order to handle the problem that the network can hardly converge effectively on large and complex datasets, a sigmoid normalize operation is applied. For multi-object tracking across cameras, we design two strategies: a simple sequence-to-sequence matching algorithm and tracking algorithm by embedding the feature extraction in EGTracker in [11]. The experiments have demonstrated that the proposed method can extract efficient features and improve the performance of tracking systems when applying the learned features. In conclusion, the contributions of this paper are as follows.

(1) NCA-Net is developed by combining deep feature learning and metric learning is constructed to acquire the high-level representation of pedestrian, aiming at improving the performance of multiple objects tracking across multiple cameras.
(2) A new loss function for NCA-Net is derived, which is more suitable for object tracking application and can resolve the convergence problem of network training.
(3) The proposed NCA-Net is applied to extract feature in two detection-based tracking system and demonstrates the favorable performance.

The remainder of the paper is organized as follows: Section 2 overviews the related work. Section 3 describes the proposed NCA-Net for pedestrian tracking. Section 4 introduces the application algorithms of the NCA-Net in multi-object tracking across multi-camera scenarios. Section 5 are the experimental results and Section 6 presents the conclusions.

## 2. Related Work

### 2.1. Tracking Framework

For the object tracking problem, the algorithm can be divided into two important components: tracking framework and feature representation. For the tracking framework, there are two main streams divided by inference methods, that is, probabilistic inference and deterministic optimization. In probabilistic inference, the states of the objects are represented as a probability distribution which is estimated by a lot of probability reasoning approaches based on the existing observations. The algorithms of probabilistic inference which are appropriate for online tracking are mainly based on Kalman filter [34], Extended Kalman filter [23] and particle filter [35]. As for deterministic optimization, it aims to obtain the maximum a posteriori (MAP) optimization to multi-object tracking (MOT), which is more suitable for off-line tracking. The deterministic optimization algorithms mainly include dynamic programming [7], min-cost/max-flow network flow [11], and conditional random field [36] and so on. Li et al. presented a temporal-spatial approach to recognize the contextual behaviors more efficiently through the event detection mechanism by combining Conditional Random Field with the results of clustering and group locating [37]. In this paper, we combine the proposed feature learning framework with two existing tracking frameworks [11,12], which are deterministic optimization and off-line algorithms.

### 2.2. Deep Metric Learning

Even though existing tracking algorithms using hand-crafted features can achieve satisfactory results [38,39], applying deep features to pedestrian tracking is a good choice to improve the performance. To make features more discriminative, metric learning is the appropriate measure when we apply deep features, due to the uncertainty of pedestrian numbers and the similarity between pedestrians. Metric learning tries to achieve the goal that the inter-class variance is maximized and the intra-class variance is minimized. The most commonly used deep metric learning strategy is based on triplet loss [40,41], which takes the triplet units as input and follows the principle that the distance of heterogeneous pair should be larger than that of the same class pair by a predefined margin. Also, some further researches adopted a larger input unit. For example, Chen et al. proposed a quadruplet loss which contained two negative samples in a single input unit [42]. Batchelor et al. adopted NCA loss in their object recognition task [43], but there is a serious problem of training difficulty, failing to converge and generalizing very badly on large scale scenes. This problem is also very common for different deep metric learning strategies. In this paper, we combine the neighborhood components analysis [44] and CNN, which can input samples of any number and constructs the loss with a probability form.

## 3. NCA-Net for Multi-Object Tracking

### 3.1. Motivation

Multi-object tracking across multiple cameras is usually divided into two steps. The first step is to match the objects in adjacent frames to form the tracklets under one camera. The second step is to connect the tracklets across cameras through tracklet matching. For both steps, we need to calculate the similarity according to the feature distance. Especially in the application of pedestrian tracking the objects may take on the similar appearance. Therefore, both the discriminative feature and distance measure are very critical. Metric learning is the general strategy to learn a suitable distance calculation formula with the training samples. On the other hand, deep learning is a practical solution to extract feature by nonlinear mapping. Goldberger et al. presented the Neighborhood Components Analysis (NCA) [43] algorithm to learn a Mahalanobis distance to improve the performance of KNN classifier. Inspired by NCA and deep learning, we propose NCA-net by combining feature learning and metric learning under a CNN framework.

### 3.2. The Structure of NCA-Net

**Figure 1:**
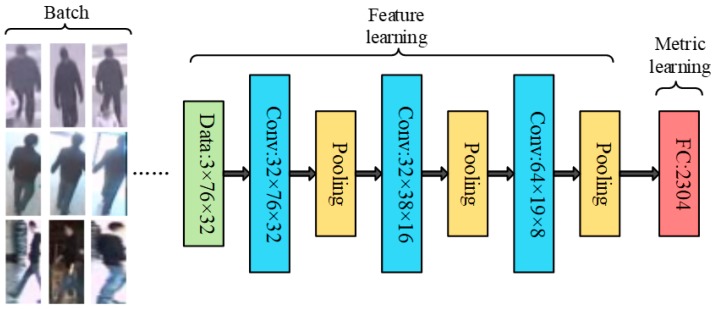
The structure of NCA-net.

The structure of NCA-net, which is experimentally implemented within the Caffe framework, is illustrated in Figure 1 [45]. NCA-net contains seven layers, including three convolution layers, three pooling layers and an inner-product layer without bias term. As we can see, NCA-net is divided into two parts: the first one extracts the feature of an object and the second part represents the computation of distance metric.

We import a batch of images into the network, in which the images from same pedestrians have same labels and different ones have different labels, and all the images are normalized to 32 × 76 (It’s for the samples from NLPR_MCT dataset. As for videos with different resolution, the size can be adjusted to suitable one). Through convolution and pooling layers, we can extract the features of ith object which appears as 2304-dimension vector $x_{i}$. Then the parameters of the final inner-product layer can form a matrix *A* that has a dimension of 2304 × 2304. Via the operation of network, we get the 2304-dimension vector *Ax_i_*. Finally, the parameters of NCA-net are optimized through the loss function according to sample distance.

For the feature learning, we adopt only three interleaved convolution and pooling layers considering that objects are normally small in the multiple cameras pedestrian tracking, and we can get the feature $x_{i}$ from object *i*. As to metric learning, we primordially aim at learning a Mahalanobis distance metric, which is generally represented by the symmetric positive semi-definite matrices. The metric that needs to study is expressed as *Q* = *A^T^A*, then the distance of *x_i_* and *x_j_* can be described as follows:

(1) $$\begin{array}{cc} d\left(x_{i},x_{j}\right) & =\sqrt{{\left(x_{i}-x_{j}\right)}^{T}Q\left(x_{i}-x_{j}\right)}\\ & =\sqrt{{\left(Ax_{i}-Ax_{j}\right)}^{T}\left(Ax_{i}-Ax_{j}\right)}\; \end{array}$$

Traditionally, what we need to learn is the matrix *A* that has the same dimension as features. Then the Mahalanobis distance of $x_{i}$ and $x_{j}$ can be replaced by the Euclidean distance of $Ax_{i}$ and $Ax_{j}$. Therefore, the matrix *A* is explicitly embedded in the FC layer.

### 3.3. Loss Function of NCA-Net

To learn the Mahalanobis distance metric, we could adopt the similar loss function as NCA which is described in Equation (3):

(2) $$p_{ij}=\frac{exp\left(-{\left|\left|D_{ij}\right|\right|}^{2}\right)}{\sum_{k\neq i}exp\left(-{\left|\left|D_{ik}\right|\right|}^{2}\right)}$$

(3) $$J_{NCA}=-\sum_{i}p_{i}=-\sum_{L_{i}=L_{j}}p_{ij}$$

where $p_{ij}$ represents how much confidence when we judge that object i and object j are from the same class, and $p_{i}$ means the probability of that object *i* will be correctly classified ($L_{i}=L_{j}$ means object *j* is in the same class as i). In the formula $D_{ij}$ indicates Euclidean distance between features $Ax_{i}$ and $Ax_{j}$ that are obtained from the last layer of NCA-net. According to the above explanations, Equation (3) suggests that the probability of sample pair from the same class being classified as the same label should be as high as possible.

However, there are far more inter-class sample pairs than the intra-class ones in the actual application, we make an alteration to involve the inter-class samples to the loss computation, which is illustrated in Equation (4):

(4) $$J=\sum_{i}\sum_{L_{i}\neq L_{j}}p_{ij}$$

From Equation (4), we can conclude that those inter-class sample pairs tend to have low probability of being labeled as the same class. In addition, both the loss function *J* ad *J_NCA_* can effectively drive training when the feature’s dimension is small and the samples are simple. However, they reach a bad value quickly (Just a few iterations) in our experiments for the reason that our task is very difficult for more than a thousand feature dimension. If we want our loss function to be effective, we have to limit the value of ${\left|\left|D_{ij}\right|\right|}^{2}$ to a relatively small number, because that $exp\left(-{\left|\left|D_{ij}\right|\right|}^{2}\right)$ is equal to zero in computer when ${\left|\left|D_{ij}\right|\right|}^{2}$> 745 (in Matlab). We denote *Ax* as $x_{i}^{*}$. During back propagation, the gradient of $x_{i}^{*}$ obtained from the last layer is as follows:

(5) $$\frac{\partial J}{\partial x_{i}^{*}}=2\sum_{L_{i}\neq L_{j}}\frac{\sum_{k\neq i}\left(x_{i}^{*}-x_{k}^{*}\right)\exp\left(-{\left|\left|D_{ik}\right|\right|}^{2}\right)-\left(x_{i}^{*}-x_{j}^{*}\right)\sum_{k\neq i}\exp\left(-{\left|\left|D_{ik}\right|\right|}^{2}\right)}{{\left(\sum_{k\neq i}\exp\left(-{\left|\left|D_{ik}\right|\right|}^{2}\right)\right)}^{2}}$$

So the gradient will disappear when the value of ${\left|\left|D_{ij}\right|\right|}^{2}$ is very large. To make the distance be a small value, we apply a sigmoid function to normalize x*, and then we calculate the distance between features $x_{i}^{*}$ and $x_{j}^{*}$ as follows:

(6) $${\left|\left|D_{ij}\right|\right|}^{2}={\left|\left|sigmoid\left(x_{i}^{*}\right)-sigmoid\left(x_{j}^{*}\right)\right|\right|}^{2}$$

The sigmoid function can limit the output value of inner-product layer to (0,1), so ${\left|\left|D_{ij}\right|\right|}^{2}$ is smaller than dimension of x*. Furthermore, sigmoid function can hide many dimensions of x* when we calculate the ${\left|\left|D_{ij}\right|\right|}^{2}$ for two large positive values or negative values even if their values vary widely, the distance between them comes to zero after a sigmoid function. In other words, the sigmoid function could greatly increase the sparseness of features for distance calculation as well as improve the representation ability of feature.

### 3.4. Training of NCA-Net

**Figure 2:**
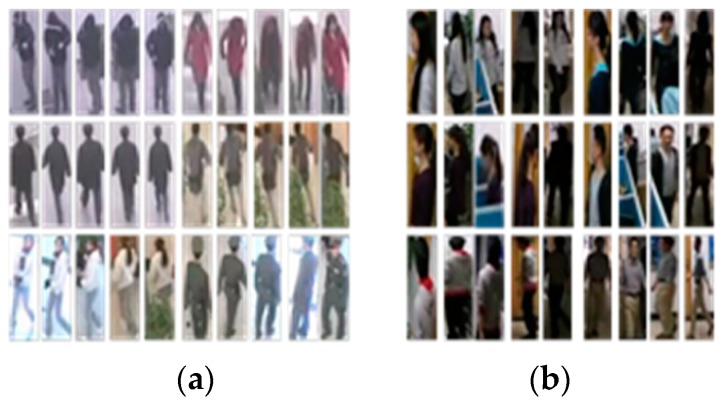
Some examples of the training data. The images in (**a**) are from subset1 of NLPR_MCT dataset, and images in (**b**) are from subset3 of NLPR_MCT dataset.

For the network training, we collect 6944 images of 117 pedestrians which are all normalized to the size of 76 × 32 pixels. The sample images are collected from NLPR_MCT dataset [8]. Some training samples are listed in Figure 2.

When training the NCA-net within Caffe framework, we take the “step” learning-rate with a “base_lr” of 0.00008, and the “stepsize” is set to 5000. For every iteration during training, we choose four pedestrians at random of which we pick out five samples randomly, then import the 20 images into data layer as a batch. In addition, we start training from the parameters that come from a pre-training in Cifar10 dataset. We validate the network each 1000 iterations and “test_iter” is set to 1000.

**Figure 3:**
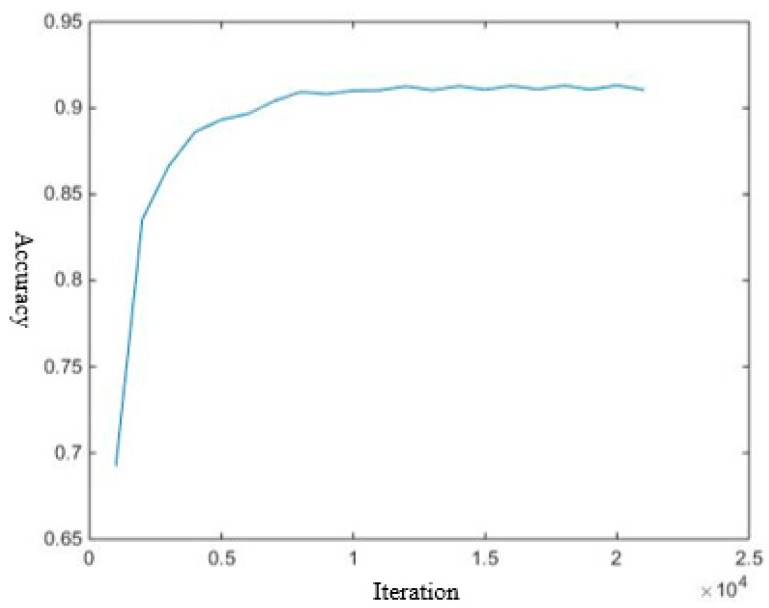
The change curve of accuracy according to iteration during training process of NCA-net.

Since our objective is to train a network to find a metric for the purpose that the sample and its closest neighbor have the same label, we define accuracy to describe the performance of NCA-net during training. By comparing if the labels of each sample and its nearest neighbor are same, we calculate accuracy which can intuitively indicate how well the network is currently trained. Accuracy (*Acc*) is expressed as:

(7) $$Acc=\frac{\sum_{i}I\left(l_{i}=l_{\left(nn\left(i\right)\right)}\right)}{N}$$

where *I* is indicator function, and *l* is the label, and nn(i) is the nearest neighbor of sample *i*, and *N* is the total number of training samples. We show the learning curve in Figure 3 in terms of *Acc*. Observing that the statistical accuracy corresponding to iteration, the network converges very quickly under effective training. Finally, we get a final accuracy 0.91.

### 3.5. Visualization of Features Extracted from NCA-Net

**Figure 4:**
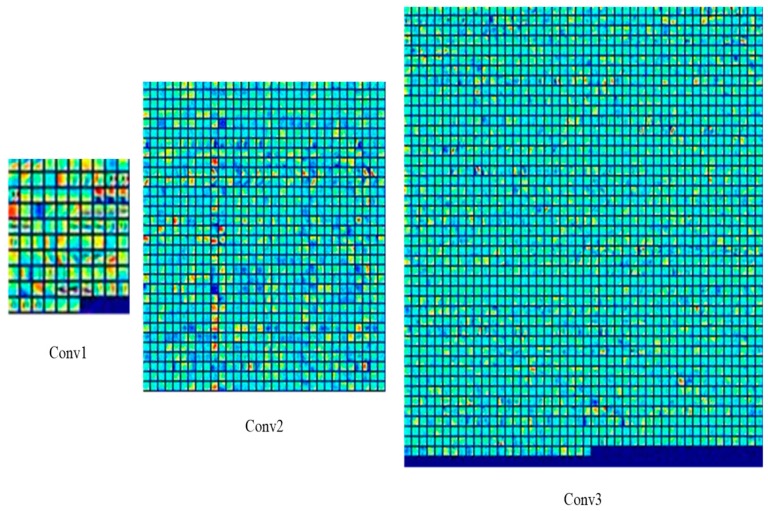
The weights visualization of NCA-net’s convolution layers.

After the training of NCA-net, we get the Caffe model, which records the weights of NCA-net that will be used in the sample matching. In Figure 4, we illustrate all the convolution kernels that used for extracting features. Generally speaking, when the patterns of convolution kernels look like noise or have a high correlation, the weights of convolution kernels are unsatisfied ones. According to Figure 4, the obtained parameter values are favorable.

**Figure 5:**
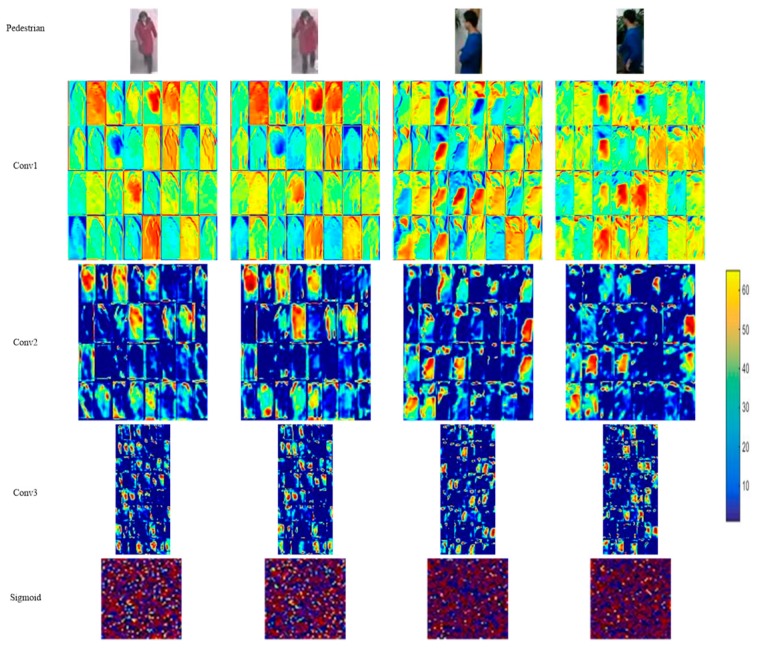
The visualization of features of two pedestrians extracted from NCA-net.

In Figure 5, we list the learned features of four samples, which belong to two pedestrians. As we can see, Conv1 focus on the different details of image, such as the outline of object and background. In Conv2 and Conv3, the feature maps are sparse and pay more attention to local areas, so that they can extract important features of the target with less irrelevant information. If we take the sigmoid normalized feature as the feature as the final layer, Euclidean distance of which represents the Mahalanobis distance between the features. We show the normalized feature in the last row of Figure 5. Intuitively, the features of the same pedestrian are similar so that the distance between different pedestrians are far larger than the same one.

### 3.6. Comparison of Normalized Feature and Original Feature Extracted by NCA-Net

Because the metric learning problem is converted to as feature learning in NCA-net, the learned distance is implemented by calculating the Euclidean distance of the learned feature. As shown in Equation (6), the original feature extracted by NCA-Net is normalized by sigmoid function, which is equivalent of adding a simple sigmoid layer to the final inner-product layer. In the following section, we explain the better applicability of the normalized feature compared to the original feature extracted by NCA-net.

**Figure 6:**
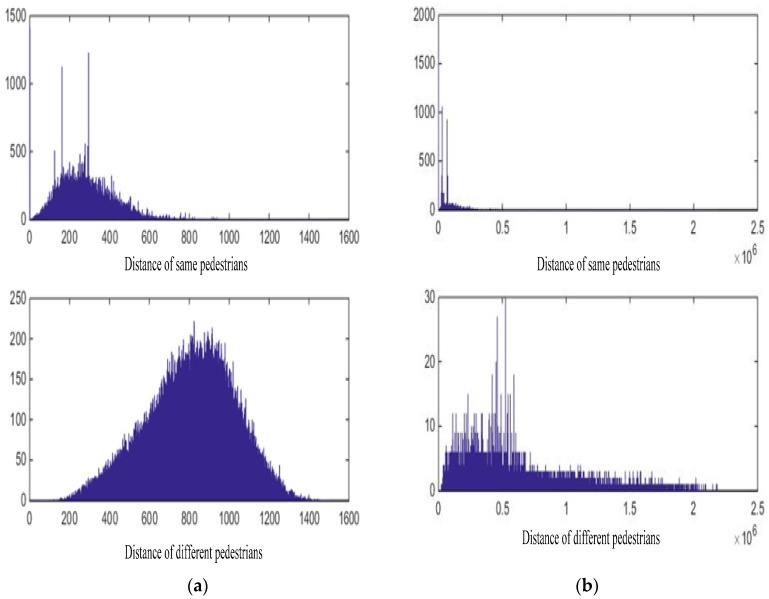
The statistics of the distance values. The features used in (**a**) are from sigmoid normalization, and features used in (**b**) are originally from inner-product layer.

To use the features in multi-target tracking of surveillance video, we can use a threshold to determine whether two objects belong to same pedestrian. We collect some samples just as what we have done when we acquire training data, and 3566 samples of 67 pedestrians were finally selected. Then we import the data into the trained NCA-net just like training, and taking outputs with a sigmoid normalization as features, then the distances between each other samples are recorded. In the end, we obtain 100,000 distance values between objects of same pedestrian, so do different ones. The statistics of distance values are displayed in Figure 6a. In addition, if we take the output of inner-product layer as features directly, we can get a statistics data like Figure 6b.

As seen in the statistics data, if we take outputs of inner-product layer as features, we can hardly determine whether two samples belong to the same object, but after sigmoid function suppresses the response of some dimension, we can achieve the objective effectively. In the end, take into account the distribution of distance values, we determine whether two samples belong to the same pedestrian as follows:

(8) $$C_{ij}=\{\begin{array}{c} 1,\;\;\;{\left|\left|D_{ij}\right|\right|}^{2}<500\\ 0,\;\;\;{\left|\left|D_{ij}\right|\right|}^{2}\geq 500 \end{array}$$

in which $C_{ij}=1$ means that object *i* and object *j* belong to the same pedestrian.

## 4. Multiple Objects Tracking Algorithm across Multi-Camera

### 4.1. Simple Match Algorithm for MCT

**Figure 7:**
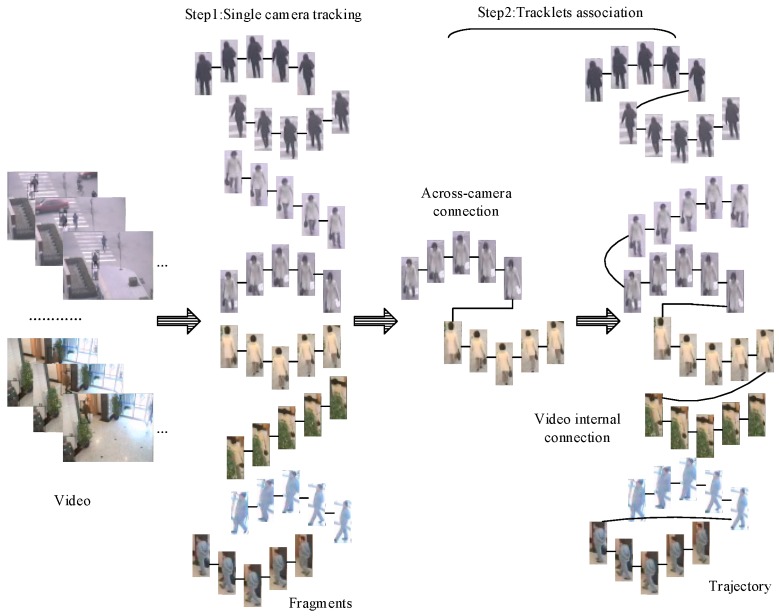
The basic processes of multi-targets tracking across multiple cameras.

When dealing with multi-target tracking across multiple cameras, we implement a simple match algorithm (SMA) as illustrated in Figure 7, and we take the result of object detection as input. Firstly, we solve single camera object tracking by a simple inter-frame target matching and then many short object sequences acquired, we associate sequences of the same pedestrian in step two.

#### 4.1.1. Single Camera Case

In multi-objects tracking in a single camera case, the appearance of objects does not have much variation between adjacent frames, and the spatial information of objects is reliable, so we simply match the targets of two adjacent frames. If an object in current frame can match another one in previous frame, we give them the same label; otherwise, we set a new label to this object. We consider two objects to be same pedestrian when they satisfy the constraints as follows:

(1) $C_{ij}=1$;
(2) Taking the video’s resolution and frame rate into consideration, space distance $d_{ij}$ needs to satisfy that $d_{ij}\leq 20$;
(3) After we finish the matching between two adjacent frames, we must avoid one pedestrian appearing twice in the same frame.

#### 4.1.2. Tracklets Association

After the multi-objects tracking in a single camera, we obtain a series of tracklets, and then we need to connect the tracklets that belong to the same pedestrian and form a complete trajectory. By exploiting the information from an image sequence, the performance of tracking algorithm could be improved. An image sequence can be obtained if we connect all the pedestrian images together when a pedestrian passes across a camera, so we may acquire the same number of pedestrian image sequences as the number of camera. If we represent a pedestrian under a camera with its image sequence, we can calculate the distance of two sequences to measure the dissimilarity of two pedestrians. However, no matter from the experimental view or the intuitive view, the mean distance is the best choice [17]. The distance between set $A=\left\{a_{1},a_{2},\ldots ,a_{n}\right\}$ and set $B=\left\{b_{1},b_{2},\ldots ,b_{n}\right\}$ can be defined as:

(9) $$S\left(A,B\right)=\frac{1}{\left|A\right|\times \left|B\right|}\sum_{i=1}^{n}\sum_{j=1}^{m}D\left(a_{i},b_{j}\right)$$

If we suppose that each sequence has the same number of images *k*, the distance of two sequences (sets) can be further expressed as:

(10) $$S\left(A,B\right)=\frac{1}{k^{2}}\sum_{a_{i}\in A}\sum_{b_{j}\in B}D\left(a_{i},b_{j}\right)$$

where S(A,B) can be replaced as $D_{ij}$ in Equation (8) to decide if the two sets are from the same pedestrian. Firstly, we associate the tracklets from different cameras, and we consider two tracklets that come from different cameras belong to same trajectory when they satisfy the constraints as follows:

(1) $C_{ij}=1$;
(2) The spatial positions of two tracklets satisfy the view field distribution of two cameras;
(3) Two tracklets do not overlap in the same time;
(4) Two tracklets do not have a time interval more than one minute because the adjacent cameras do not distribute far away;
(5) One tracklet can match no more than one tracklet.

Secondly, we associate the tracklets from the same cameras, for the fact that a complete trajectory may divided into several pieces by occlusion. Analogously, we predict two tracklets belong to the same trajectory when they satisfy the constraints as follows:

(1) $C_{ij}=1$;
(2) The connection points of two tracklets have small spatial distance, such as $d_{ij}\leq 50$;
(3) Two tracklets do not overlap in the same time;
(4) Two tracklets do not have a time interval more than five second, because occlusion always keep a short time;
(5) One tracklet can match no more than two tracklets, one before it with another after.

In the process of track correlation, we randomly select five samples of each tracklet and extract their features by NCA-net, and then we treat these features as tracklet’s features. The distance between two tracklets is represented as the average distance between the features of their five samples.

### 4.2. EGTracker Using Metric Learnt by NCA-Net

Chen et al. dealt with the multi-camera tracking (EGTracker) using a global graph model and an improved similarity metric [11]. The approach computes the similarity in different way in the caes of single camera tracking and inter-camera tracking and optimize the global graph model. Here we utilize the framework of EGTracker, but apply the features from NCA-net.

EGTracker focuses on optimizing multi-camera tracking by a global data association, which is presented as a problem of global maximum a posteriori (MAP). The MAP association problem can be optimized by a min-cost flow graph that formulated as G = {N, E, W}, where N is the node set, and E is the edge set, and W is the weight set. In the global graph model, the tracklets produced by single-object tracking are used to produce nodes N. For edges E, three rules are designed to choose the connecting edges between the tracklets. Edge weight W that is computed through appearance similarity and motion similarity in the original algorithm [8], is now obtained using the sigmoid normalized features extracted by NCA-net. We will make a comparison with the original EGTracker algorithm in the experiment section.

## 5. Experimental Results

### 5.1. Experiment on NLPR_MCT Dataset

**Figure 8:**
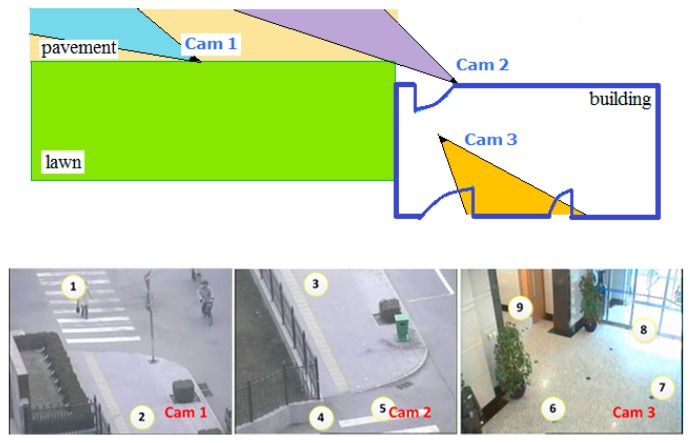
The layout of cameras of subset 1 is shown in the upper image, and the view of each camera is shown in bottom while the numbers denote entry/exit areas. This figure is adapted from: http://mct.idealtest.org/Datasets.html.

The Multi-Camera Tracking (MCT) challenge which was in conjunction with ECCV 2014 focused on the problem of object tracking across multiple non-overlapping cameras. It provided a dataset (NLPR_MCT), which includes four subsets captured from different non-overlapping multi-camera networks, as we illustrate subset 1 in Figure 8. To train NCA-net, we cut a third of each video in subset1 and two-thirds of each video in subset 3, and then we gather pedestrian samples from these video clips with an interval of five frames. Then we test algorithms on the complete NLPR_MCT dataset.

To evaluate how well the proposed algorithm performs from the aspect of pedestrian MCT, we can calculate two statistical data that contain SPMI and MPSI. SPMI represents that a single pedestrian has multiple IDs and MPSI means multiple pedestrians have the same ID. When a single pedestrian is connected with *n* IDs, SPMI value will plus *n* − 1, and the MPSI value will plus *n* − 1 while *n* different pedestrians share the same ID.

Besides, to measure the effects of our algorithm on the tracking task, we choose multi-camera object tracking accuracy (MCTA) as the evaluation index proposed in [11]. The MCTA, a modified version of MOTA [46], is expressed as follows:

(11) $$\mathrm{MCTA}=\mathrm{Detection}\times {\mathrm{Tracking}}^{\mathrm{SCT}}\times {\mathrm{Tracking}}^{\mathrm{ICT}}$$

where “Detection” represents the ability of target detection and defines as these:

(12) $$\mathrm{Dectecion}=\frac{2\times Precision\times Recall}{Precision+Recall}$$

where *Precision* is the accuracy of detection and *Recall* is the ratio that the actual targets are detected. In our experiment, we take the ground truth bounding boxes as input, so we have Detection = 1. Tracking^SCT^ and Tracking^ICT^ measure the performance of single camera and inter-camera object tracking via the number of mismatch (ID-switches), respectively. We calculate them as follows:

(13) $${\mathrm{Tracking}}^{\mathrm{SCT}}=1-\frac{\sum_{t}mme_{t}^{s}}{\sum_{t}tp_{t}^{s}}$$

(14) $${\mathrm{Tracking}}^{\mathrm{ICT}}=1-\frac{\sum_{t}mme_{t}^{c}}{\sum_{t}tp_{t}^{c}}$$

where $mme_{t}^{s}$ indicates the mismatches appear in a single camera while $mme_{t}^{c}$ represents those mismatches in inter-camera case. $tp_{t}^{s}$ and $tp_{t}^{c}$ are the matching frames in ground truth which are shown in Table 1. $tp_{t}^{s}$ contains the matching of adjacent frames that belong to single camera, and $tp_{t}^{c}$ represents the matches in the case of inter-camera.

**Figure 9:**
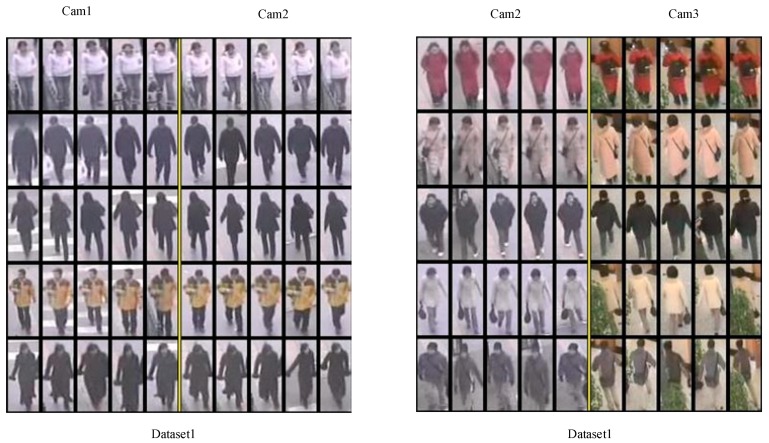

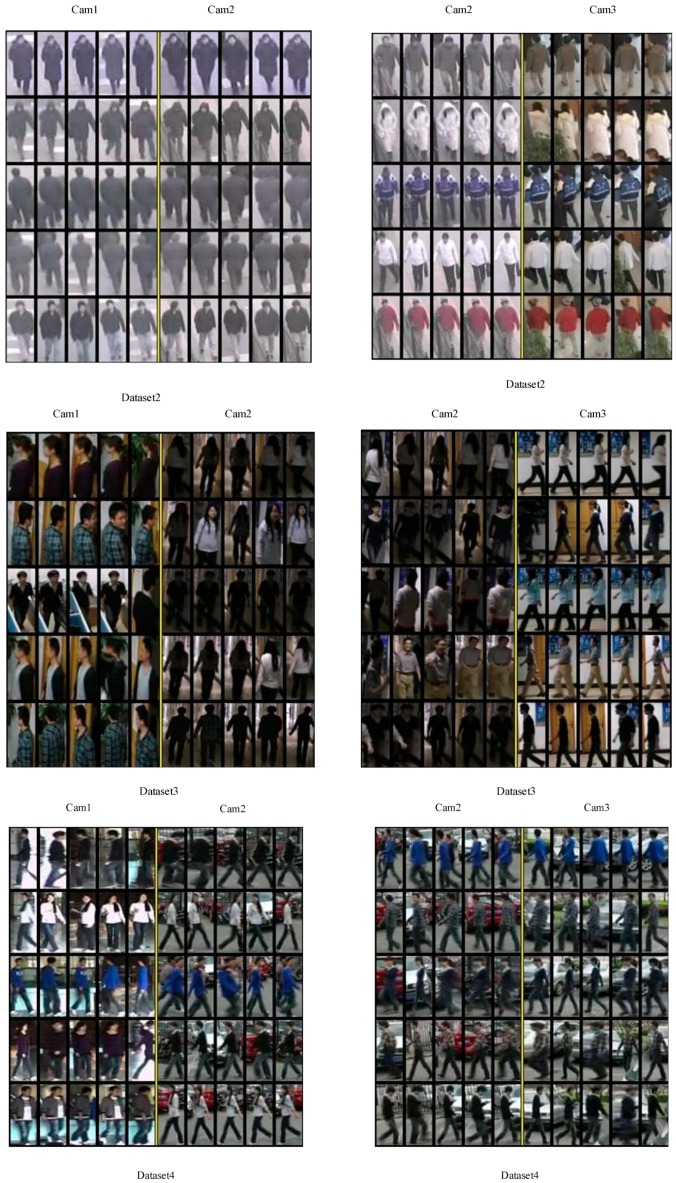
Part of the inter-camera matching results.

We test the proposed SMA in NLPR_MCT dataset. The bounding boxes of ground truth detections are taken as input without utilizing the relationship between bounding boxes (i.e., identities). Part of the inter-camera matching results are shown Figure 9. By analyzing the visual qualitative results, we may draw a conclusion that SMA performs well on the dataset1 and dataset2, whether matching between outdoor cameras or outdoor and indoor. It performs worse in dataset4 since here are several wrong pedestrians in different camera to be connected. But for dataset3, it behaves unsatisfied because many tracklets from different pedestrian are tracked together. There are two possible reasons. One is that the task itself is very difficult, such as intense illumination change and view angle variance. As we can see, the illumination in cam2 is so dark and only the upper body can be seen for objects from cam1. The second reason is that appearance feature is not competent enough. In our training configuration, only the samples of 117 pedestrians are collected from dataset1 and dataset3 for training, but only 14 of them are from dataset3. Therefore, the learned features extracted from NCA-net underfit the case of dataset3.

To quantitively evaluate performance, the statistical results of SPMI and MPSI are displayed in Table 2, and we compare with the results that come from EGTracker [11]. For MPSI, our algorithm is obviously better than EGTracker, which means the proposed method is good at matching tracklets with fewer mismatches.

As for SPMI, SMA does better in the first two datasets, but worse in the last two due to the complicated environment and less training samples. In addition, SPMI value is always far above MPSI value, which suggests we get too many fragments during single camera tracking, and we can hardly associate the fragments together.

For MCT tracking performance, we adopt MCT evaluation kit to calculate the MCTA values of our experiment results, which can be download in “http://mct.idealtest.org/Result.html”. Some results of the Multi-Camera Object Tracking (MCT) Challenge are offered in the website too. Thus, we compare SMA with several novel methods including EGTracker [11], USC_Vision [47,48], hfutdspmct (the algorithm’s results are from the website: http://mct.idealtest.org), CRIPAC_MCT [49]. The results are listed in Table 3.

As shown in Table 3, the proposed matching algorithm SMA has the highest average MACT value, which suggests we get a best performance over the entire dataset. Especially in the dataset1 and dataset2, our algorithm obviously has advantages over the other algorithms. However, in dataset3 we perform worse than the EGTracker, because our algorithm relies on the feature learning with the fact that we use the simplest way of tracking and association, and we only have training samples of 14 pedestrians from dataset3. As for dataset4, we behave a little poorer compared to USC_Vision, which is the winner in the (MCT) Challenge of ECCV 2014 visual surveillance and re-identification workshop. The main reason is possible that we don’t select any training samples from dataset4.

In above experiments, we use the deep features from NCA-net in the simplest way and compare it with the most effective methods from MCT challenge. Furthermore, combining our deep feature with the other tracking framework we can obtain the better result. As an instance, we apply the features in EGTracker which is described in Section 4.2 and identified as Ours+EGTracker. To explain clearly, the results are listed in Table 4.

Because few training samples are selected from the dataset3, the performance is slightly inferior to that of EGTracker. The experimental results show that Ours+EGTracker achieves the highest average MCTA. It demonstrates that the learnt deep features based on NCA-like metric can improve the performance of tracking system where the feature distance is the most crucial component.

### 5.2. Extended Experiment on DukeMTMC Dataset

Aiming to speed up the development of multi-target multi-camera tracking, DukeMTMC is a dataset in the Multiple Object Tracking Benchmark of MOT Challenge which was created by Ristani et al. in 2016 [12]. The DukeMTMC dataset taken by eight synchronized cameras contains over 7000 single camera trajectories and more than 2000 individuals, and each camera has recorded 85 min of 1080p video with 60 fps. The dataset is separated into one training/validation set and two test sets, which are named as test-easy and test-hard respectively. In addition, evaluation toolkit is provided by MOT Challenge and a benchmark single-camera tracking systems is available in DukeMTMC project.

**Figure 10:**
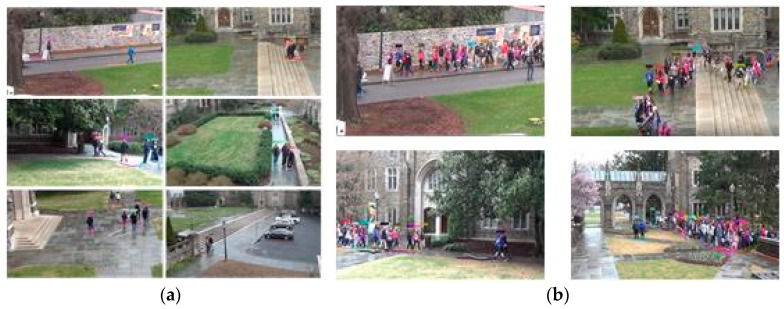
Some fragments of single-camera tracking. The results from Cam1~Cam8 are showed above. Subfigure (**a**) shows the trajectories of the test-easy dataset and subfigure (**b**) displays the obtained trajectories from the test-hard dataset.

In the experiment, we choose the detection provided by dataset as the input of the multi-object tracking. Since there are only detection results of test sets being provided, and more than 90% of the detections are background boxes, we filter the obvious background detections before tracking. Then we extract features of the filtered detections and apply them to the single-camera tracking system which is provided by DukeMTMC project [12]. Some fragments of the result are showed in Figure 10.

As we can see in Figure 10a, the tracker behaves well when the pedestrian overlap is not serious. However, as an example of test-hard dataset, in the case that a group of students walk together after class, there are 60 people walking across the field the tracker can only hold several of objects. As seen in Figure 10b, the tracker can only hold several of objects, such as only seven targets in the top-left image, eight targets in the top-right and bottom-left images hold successfully, and 12 trajectories obtained in the bottom-right image. Therefore, our method is most suitable for situations where the number of targets is large and the target state changes greatly but the mutual occlusion is not seriously. To measure the algorithm performance, we select five representative indicators from the official evaluation toolkit. The indicators include IDF1, IDP, IDR, MOTA and MOTP [12,46]. IDF1 means the ratio of the recognized detections over the average number of ground-truth and the actual predicted detections. IDP represents identification precision which is the fraction of computed detections that are correctly identified and IDR means identification recall. Three error sources of multiple object tracking accuracy (MOTA) are combined: false positives, missed targets and identity switches. Multiple object tracking precision (MOTP) embodies the mismatch between the labeled and the computed bounding boxes. The higher the five indicators are, the better is the performance.

**Figure 11:**
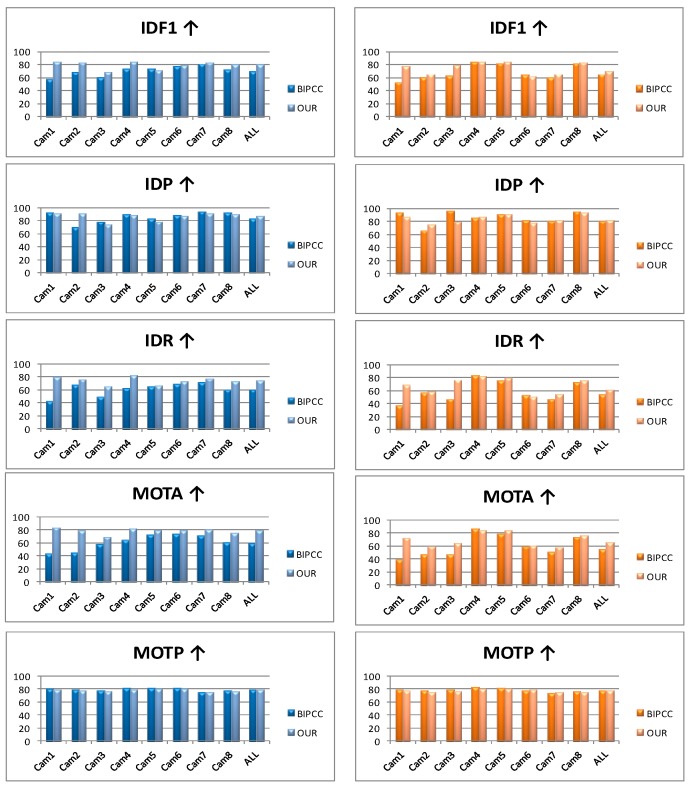
Comparison between the benchmark and ours. The IDF1, IDP, IDR, MOTA and MOTP arranged from top to bottom, and the bar graphs on the left are the results of test-easy set, the rights are the results of test-hard set.

Then we perform a comparison between our results and the benchmark [12] with the five evaluation indicators. As illustrated in Figure 11, combining our deep features with the single-tracking system performs better in IDF1, IDR and MOTA, but little down in IDP and MOTP which rely much on detection precision. Although we have filtered out most of the background, there are still many remnants, especially in Cam3. In addition, there are more than 100,000 frames of each camera in test sets, and we have to split them into many pieces considering the computing power, so there are some additional ID switches.

### 5.3. Discussion

Since network is not very complicated, all the algorithms are implemented in a desktop computer with the configuration: 16G memory, Intel Core i7-7700k CPU and a NVIDIA GTX 1080 GPU.

We present NCA-Net to extract the features for the multi-object tracking in order to better represent the discrimination of the objects. As a feature representation approach, it could be embedded into different tracking strategies. Even though it can strengthen the ability of the tracker to distinguish the different objects which are close in appearance, NCA-Net needs enough samples to train the model. If the resolution of a pedestrian is enough, like above 76 × 32 pixels, some pedestrians that are easy to be recognized as the same one can be distinguished by the proposed feature representation, such as the second and the third rows of the results of dataset1(cam1 and cam2), and the first and the second row of the results of dataset2(cam1 and cam2) in Figure 9. However, it performs unfavorable when the training samples are not enough. For example, it behaves poorer compared to USC_Vision for dataset4. From the results of dataset3 and dataset4 in Figure 9, we can also find that there are more mismatches than that of dataset1 and dataset2.

In the extended experiments on DukeMTMC dataset also show that NCA-Net can only achieve little improvements when the scenarios exists serious occlusion, like the case of the test-hard dataset.

Therefore, the proposed feature extraction approach for multi-object tracking across multi-camera has some limitations: (1) the best performance needs enough training samples; (2) the image size of pedestrian should be big enough, like over 76 × 32 pixels; (3) it has little improvement over performance when there is serious pedestrian occlusion.

## 6. Conclusions

In this paper, we propose to extract the deep features by NCA-net that combine the idea of NCA and CNN structure, and also present a strategy that solves the training difficulty in convergence problem. We apply the NCA-net in two datasets to extract appearance features of pedestrians. The results in NLPR_MCT dataset shows that some simple constraints coupled with our deep features can deal with MCT problem well, and it performs better when we integrate it to a more complex tracking model. As for the larger scale DukeMTMC dataset, the benchmark single-tracking system wins the improvement of overall performance when we replace the appearance features with the proposed deep feature. Therefore, combining the deep features with these subtly designing tracking systems can effectively relieve the performance loss caused by artificial appearance feature.

## Figures and Tables

**Table 1:** Ground truth for all four sub-datasets.

**Dataset1**		**Dataset2**	
**Tracking^SCT^**	**Tracking^ICT^**	**Tracking^SCT^**	**Tracking^ICT^**
71,853	334	88,419	408
**Dataset3**		**Dataset4**	
**Tracking^SCT^**	**Tracking^ICT^**	**Tracking^SCT^**	**Tracking^ICT^**
18,187	152	42,615	256

**Table 2:** The statistical result of spmi and mpsi.

	SMA		EGTracker	
Data	SPMI	MPSI	SPMI	MPSI
dataset1	66	6	94	42
dataset2	111	10	144	97
dataset3	171	34	78	67
dataset4	212	43	195	126

**Table 3:** Performance comparison when input is ground truth of object detection.

		SMA	EGTracker	USC_Vision	hfutdspmct	CRIPAC_MCT
1	$mme_{t}^{s}$	44	66	63	77	135
	$mme_{t}^{c}$	24	49	35	84	103
	MCTA	0.9276	0.8525	0.8831	0.7477	0.6903
2	$mme_{t}^{s}$	66	93	61	109	230
	$mme_{t}^{c}$	49	107	59	140	153
	MCTA	0.8792	0.7370	0.8397	0.6561	0.6234
3	$mme_{t}^{s}$	93	51	93	105	147
	$mme_{t}^{c}$	89	80	111	121	139
	MCTA	0.4124	0.4724	0.2427	0.2028	0.0848
4	$mme_{t}^{s}$	73	128	70	97	140
	$mme_{t}^{c}$	152	159	141	188	209
	MCTA	0.4056	0.3778	0.4357	0.2650	0.1830
Average MCTA		0.6562	0.6099	0.6003	0.4679	0.3954

**Table 4:** Comparison results of three algorithms.

		EGTracker	SMA	Ours+EGTracker
Dataset1	$mme_{t}^{s}$	66	44	64
	$mme_{t}^{c}$	49	24	21
	MCTA	0.8525	0.9276	0.9363
Dataset2	$mme_{t}^{s}$	93	66	93
	$mme_{t}^{c}$	107	49	51
	MCTA	0.7370	0.8792	0.8741
Dataset3	$mme_{t}^{s}$	51	93	80
	$mme_{t}^{c}$	80	89	83
	MCTA	0.4724	0.4124	0.452
Dataset4	$mme_{t}^{s}$	128	73	140
	$mme_{t}^{c}$	159	152	138
	MCTA	0.3778	0.4056	0.4594
Average MCTA		0.6099	0.6562	0.6805
